# Supplementary material for: Oceanic upper crustal accretion by melt sill and lava flow interaction at Axial volcano
Source: Nat Commun. 2026 Mar 5;17:3512. doi: 10.1038/s41467-026-70033-x (PMC13084056; doi:10.1038/s41467-026-70033-x)
Supplement: Supplementary file 1 — Supplementary Information [file 41467_2026_70033_MOESM1_ESM.pdf]

Supplementary Information

Oceanic upper crustal accretion by melt sill and lava flow interaction at Axial volcano

Han Wu[1,2], Wenxin Xie[1], Satish C. Singh[1*], Hélène Carton[1], Graham M. Kent[1,3], Adrien F. Arnulf [4], and Alistair J. Harding[5]

1 Université Paris Cité, Institut de Physique du Globe de Paris, CNRS, UMR 7154, F-75005, Paris, France.
2 Sun Yat-sen University, School of Earth Sciences and Engineering, 519082, Zhuhai, China.
3 Nevada Seismological Laboratory, MS-0174, University of Nevada, Reno, Reno, NV 89557 USA
4 Amazon, San Diego, CA, USA
5 Cecil H. and Ida M. Green Institute of Geophysics and Planetary Physics, Scripps Institution of Oceanography, University of California San Diego, La Jolla, CA 92093, USA

* Corresponding author (Satish Singh, singh@ipgp.fr)

This file includes:

Supplementary Figures 1-24

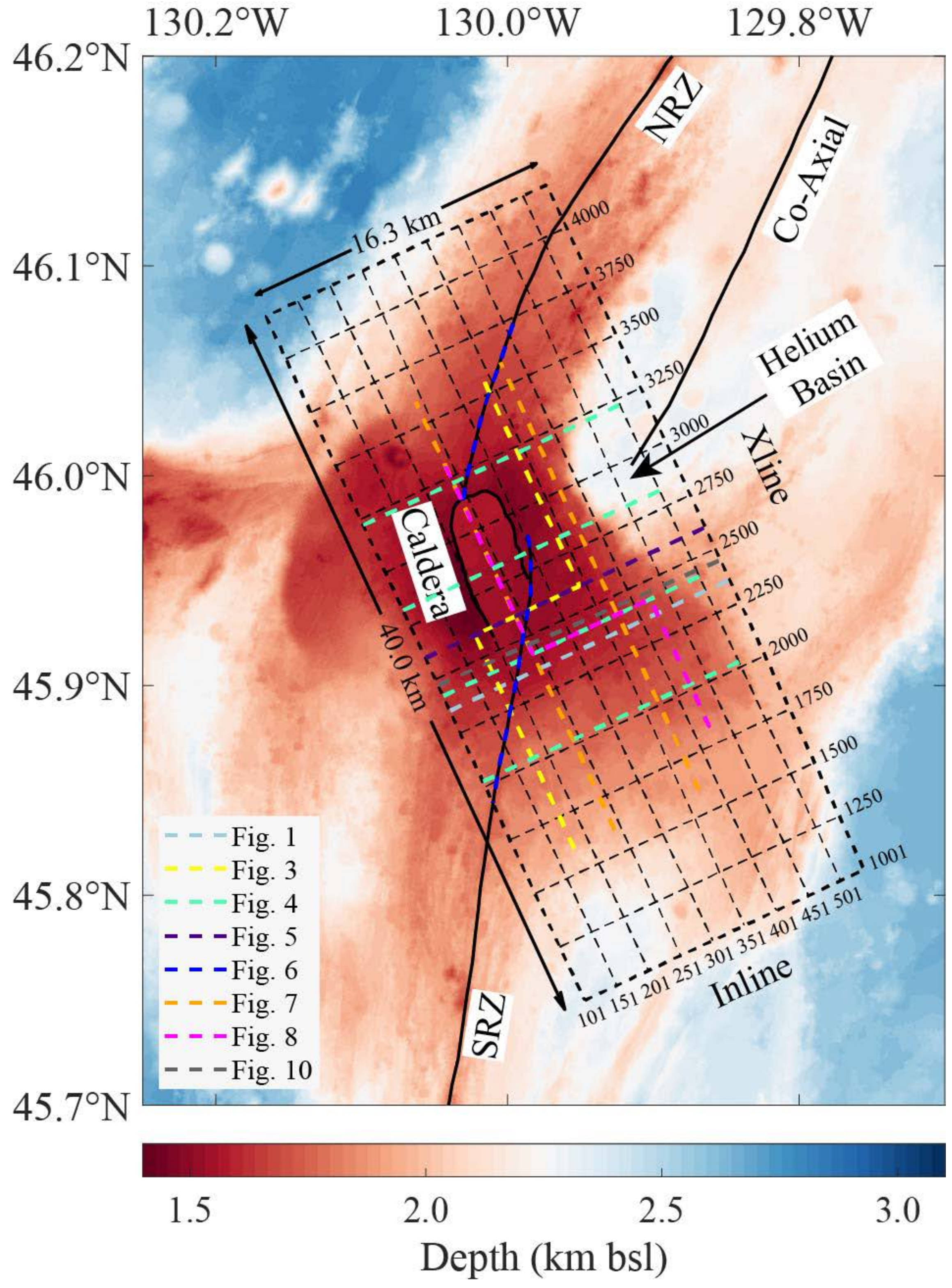

Supplementary Figure 1**. The locations of all profiles used in the main text.** A bathymetric map of the Axial volcano area, showing the north rift zone (NRZ), south rift zone (SRZ), and Co-Axial segment of the Juan de Ridge. The thick black dashed rectangle indicates the area of the 3D seismic reflection box and thin black dashed lines mark the Inline and Xline numbers. The dash colourful lines indicate the seismic lines used in the main Figures and Supplementary Figures. The dashed lines and associated figure numbers are shown in the lower left corner.

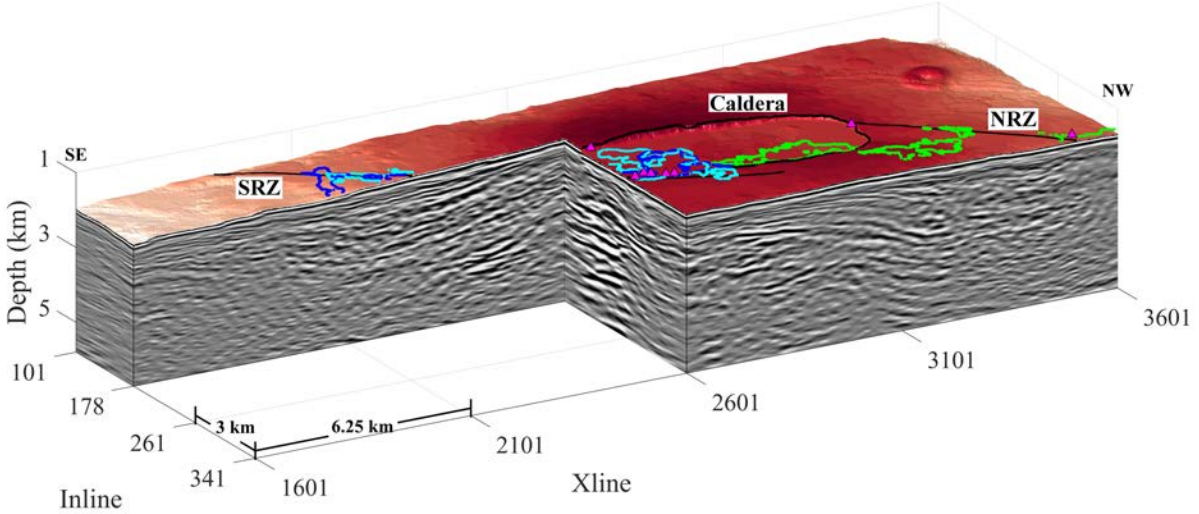

Supplementary Figure 2**. Lava flows (non-interpreted Figure 3).** A block diagram showing Inline (178 in the back and 357 at the front) seismic reflection images crossing the south rift zone (SRZ) and north rift zone (NRZ) and the south end the summit caldera. Locations of hydrothermal fields are shown as small, magenta-filled triangles[1,2]. Outlines of the 1998 (blue), 2011 (cyan) and 2015 (green) eruption lava flows are overlain on the bathymetric map[3-5].

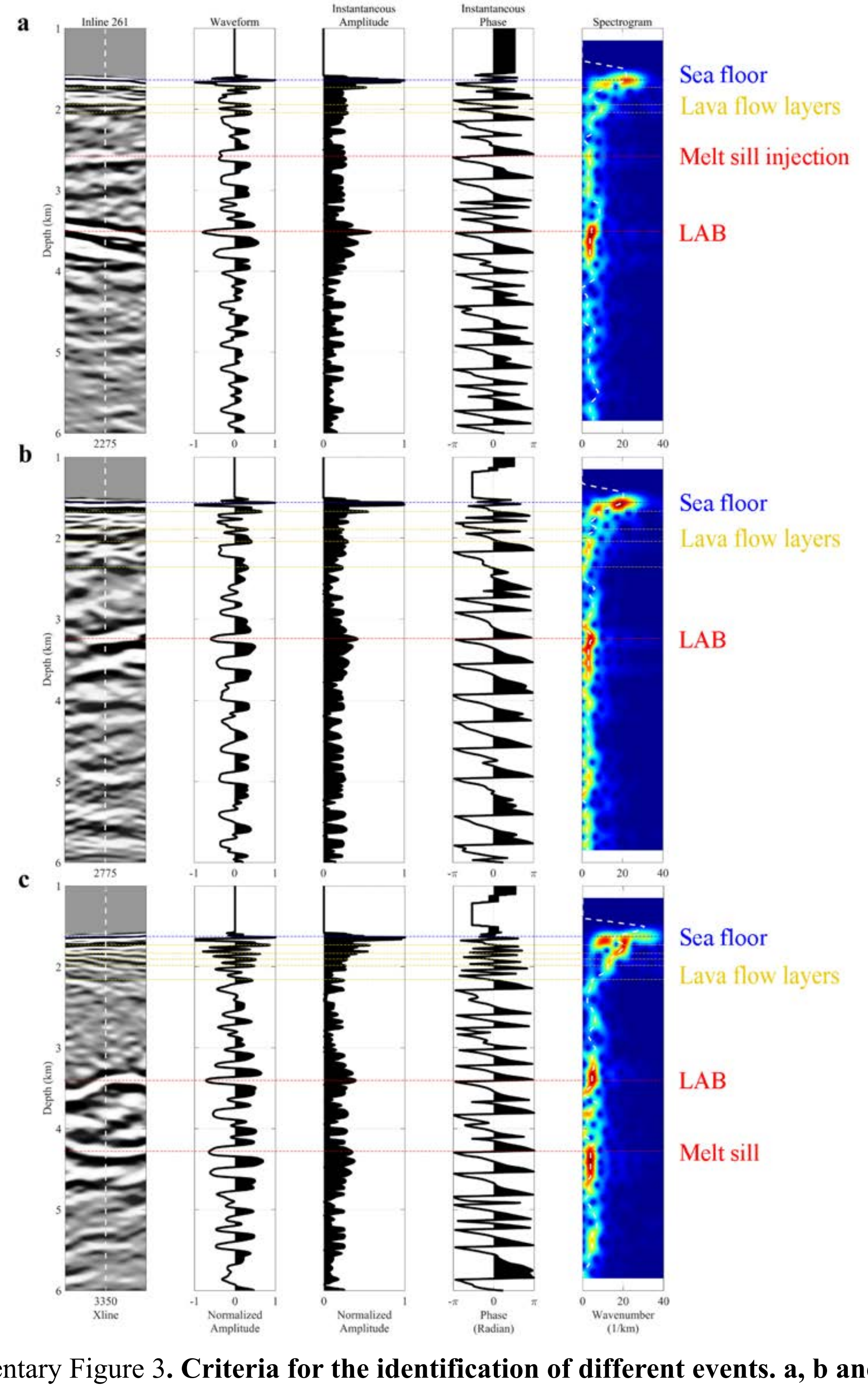

Supplementary Figure 3**. Criteria for the identification of different events. a, b and c** show the local seismic images, waveforms, instantaneous amplitudes, instantaneous phases and spectrogram at Xlines 2275, 2775 and 3350, respectively, along Inline 261. The location of different events is emphasized using the blue (seafloor), yellow (lava flow layers) and red (melt sills). LAB: Lithosphere-Asthenosphere Boundary. White dashed lines in the spectrogram indicate the wavenumber with the highest energy at different depth. See the Methods section for the detailed description.

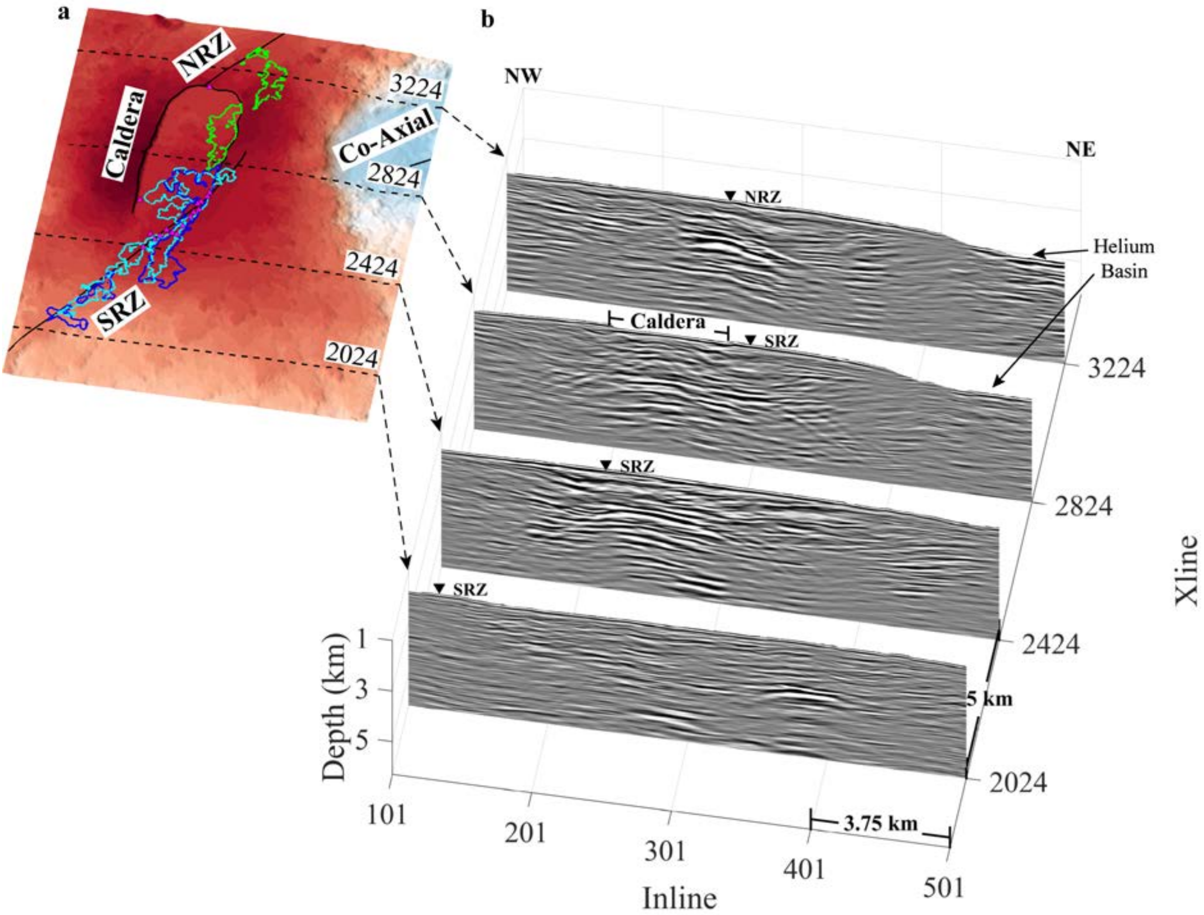

Supplementary Figure 4**. Selected Xlines from the 3D volume (non-interpreted Figure 4):** Four representative Xlines (5 km apart) showing lava flow layering, melt lenses and faults. NRZ: north rift zone, SRZ: south rift zone. The locations of the lines are shown on the map (left). Xlines 2024 and 2424 are southeast of the caldera, Xline 2824 crosses the caldera in the middle and Xline 3224 is northwest of the caldera. Locations of hydrothermal fields are shown as small, magenta-filled triangles[1,2]. Outlines of the 1998 (blue), 2011 (cyan) and 2015 (green) eruption lava flows are overlain on the bathymetric map[3-5].

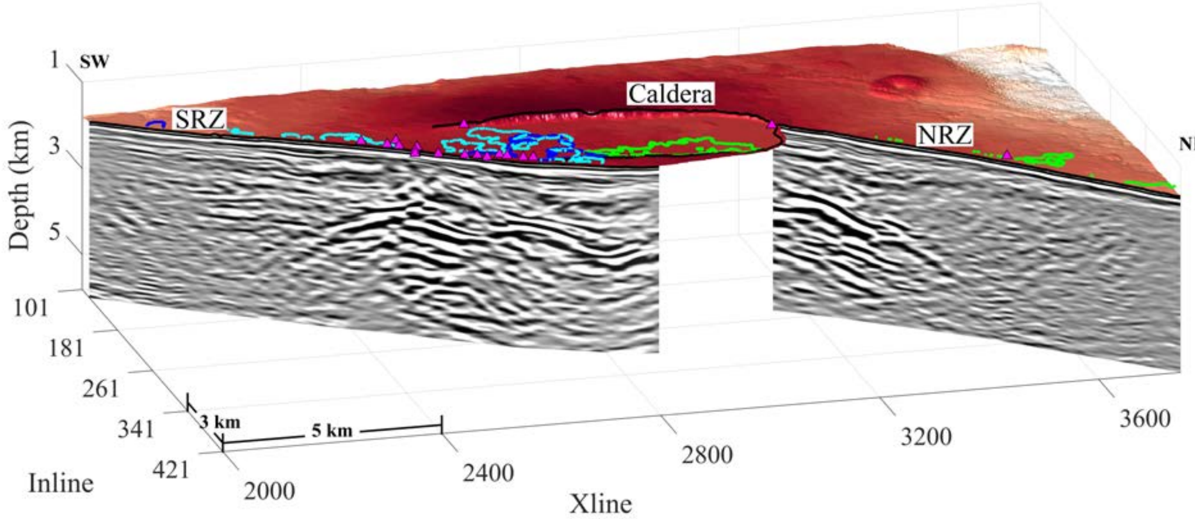

Supplementary Figure 5**. Seismic images along rift zones (non-interpreted Figure 6).** Rift-coincident seismic images along the north (NRZ) and south rift zones (SRZ) (light blue lines in Supplementary Figure 1). Locations of hydrothermal fields are shown as small, magenta-filled triangles[1,2]. Outlines of the 1998 (blue), 2011 (cyan) and 2015 (green) eruption lava flows are overlain on the bathymetric map[3-5].

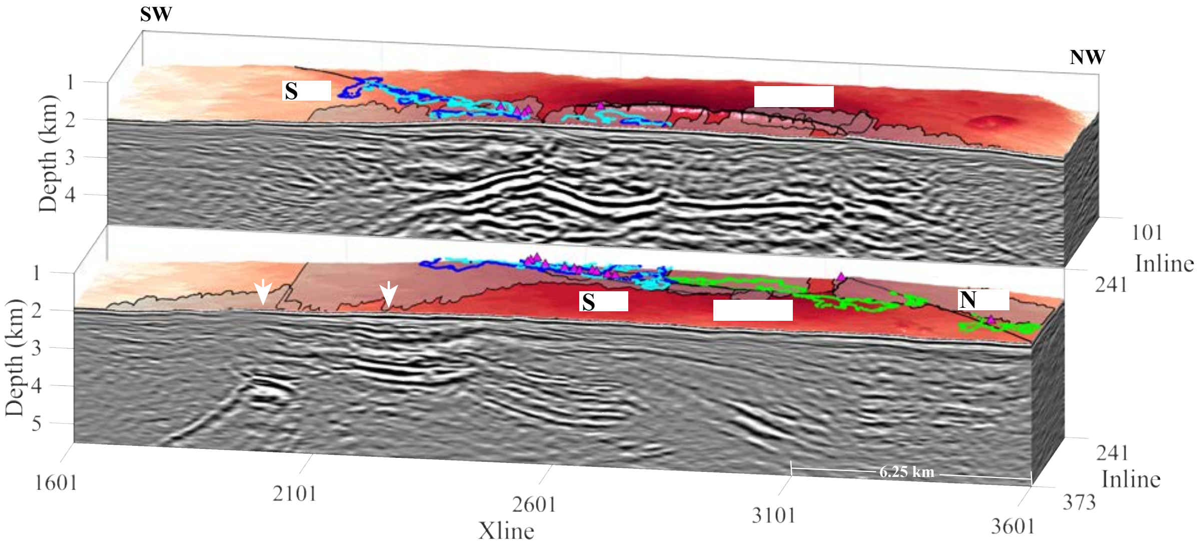

Supplementary Figure 6. **Lava flow–melt sill interaction (non-interpreted Figure 7).** Block diagram showing Inline (241 in the back and 373 in the front) seismic reflection images. White arrows indicate the two melt sills reported by Kent et al.[6]. Locations of hydrothermal fields are shown as small, magenta-filled triangles[1,2]. Outlines of the 1998 (blue), 2011 (cyan) and 2015 (green) eruption lava flows are overlain on the bathymetric map[3-5].

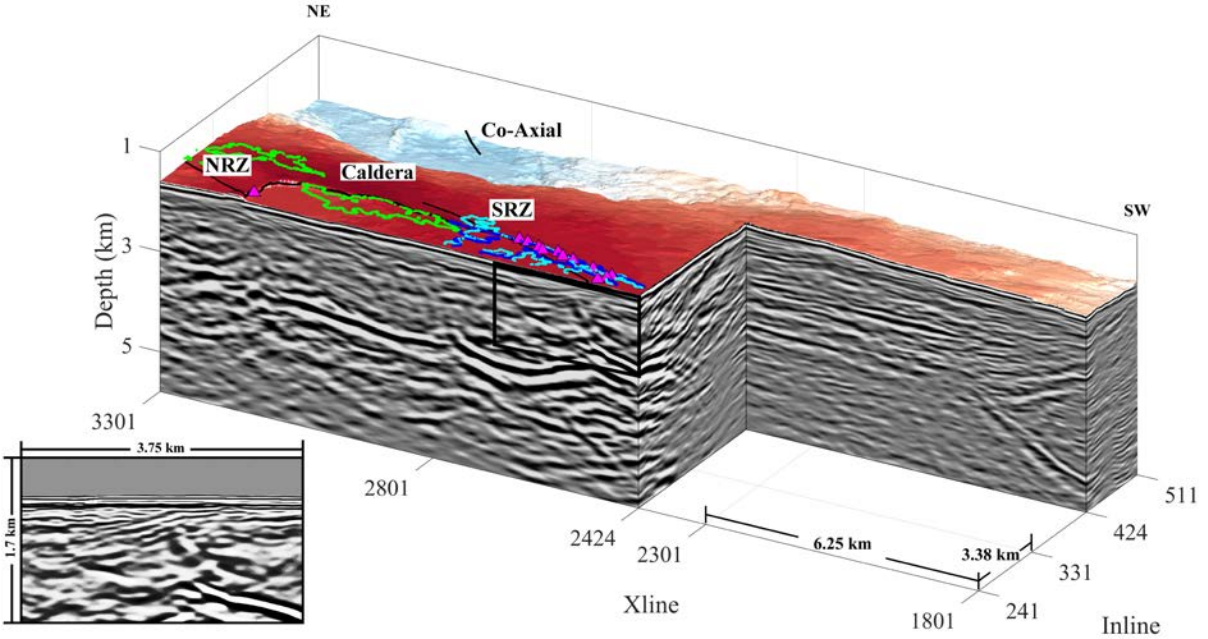

Supplementary Figure 7**. Melt sills injected along lava flow layers (non-interpreted Figure 8).** Block diagram showing Inline (241 in the front and 424 in the back) and Xline (2424) seismic reflection images. The location of inset (bottom left corner) is shown by black box in upper right of Inline 241. Locations of hydrothermal fields are shown as small, magenta-filled triangles[1,2]. Outlines of the 1998 (blue) and 2015 (green) eruption lava flows are overlain on the bathymetric map[5].

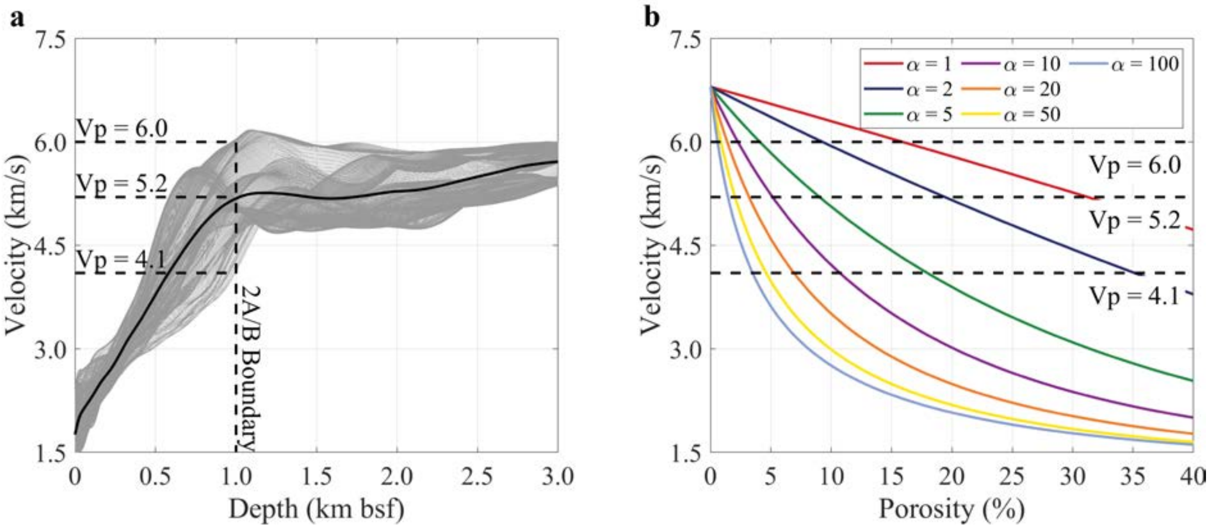

Supplementary Figure 8. **Velocity-depth profile and velocity-porosity relationship. a** 1D velocity curves with the grey solid lines representing 1D velocity structures at various locations in the inverted velocity model (see below), and the black solid line is the average 1D velocity model. The vertical black dashed line indicates the depth of the conventional Layer 2A/B boundary, associated with the change in the velocity gradient. The horizontal dashed lines indicate the minimum, average and maximum P wave velocities at the Layer 2A/2B boundary. **b** Velocity-porosity relationships calculated using the differential effective medium (DEM)[7], where α in the inserted legend indicates the inverse aspect ratio. Black dashed lines indicate the velocities at the conventional Layer 2A/B boundary.

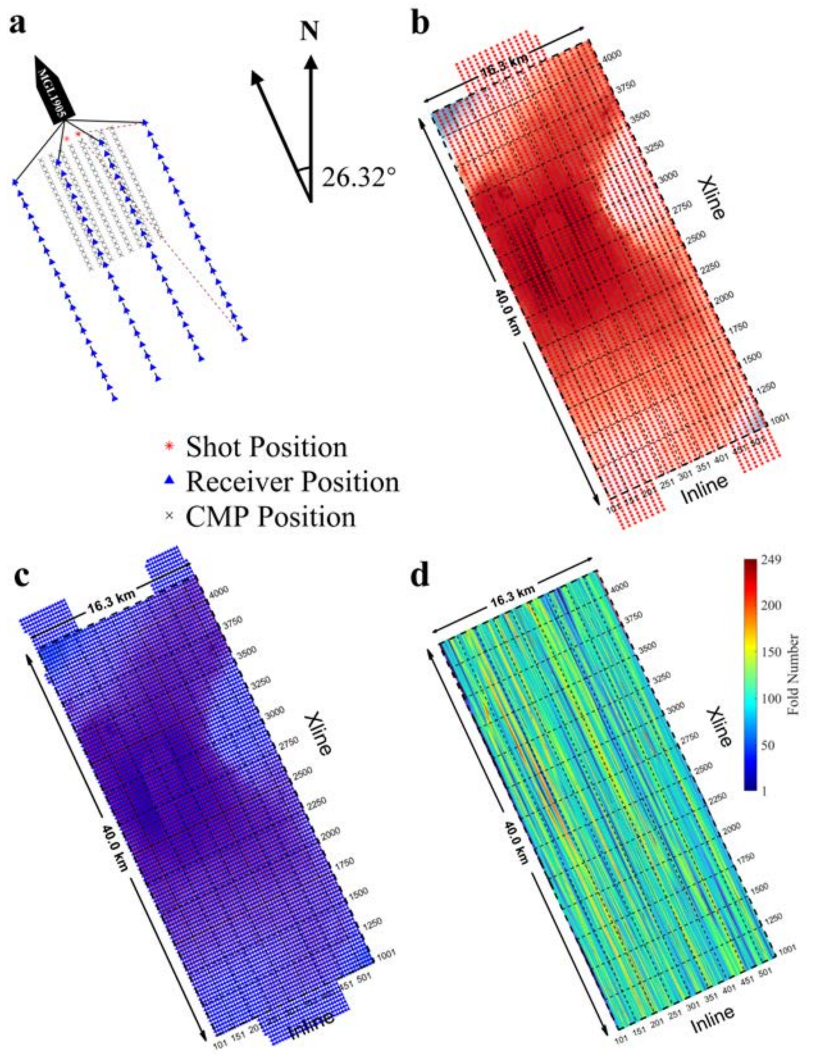

Supplementary Figure 9**. The geometry of the 3D survey[6]. a** detailed map of a linear arrangement of receivers and sources of the seismic survey distribution. **b** shot positions, **c** receiver positions, **d** fold numbers (number of traces in each bin of size 37.5 m x 12.5 m), CMP: common midpoint. The ideal distribution of midpoints includes the acquisition of two sources (75 m apart) and four streamers (150 m apart). The red asterisks and blue triangles represent the locations of the seismic sources and receivers, which are typically arrays of hydrophones towed behind survey vessels. The black crosses marked midpoints indicate the locations halfway between each source and receiver pair.

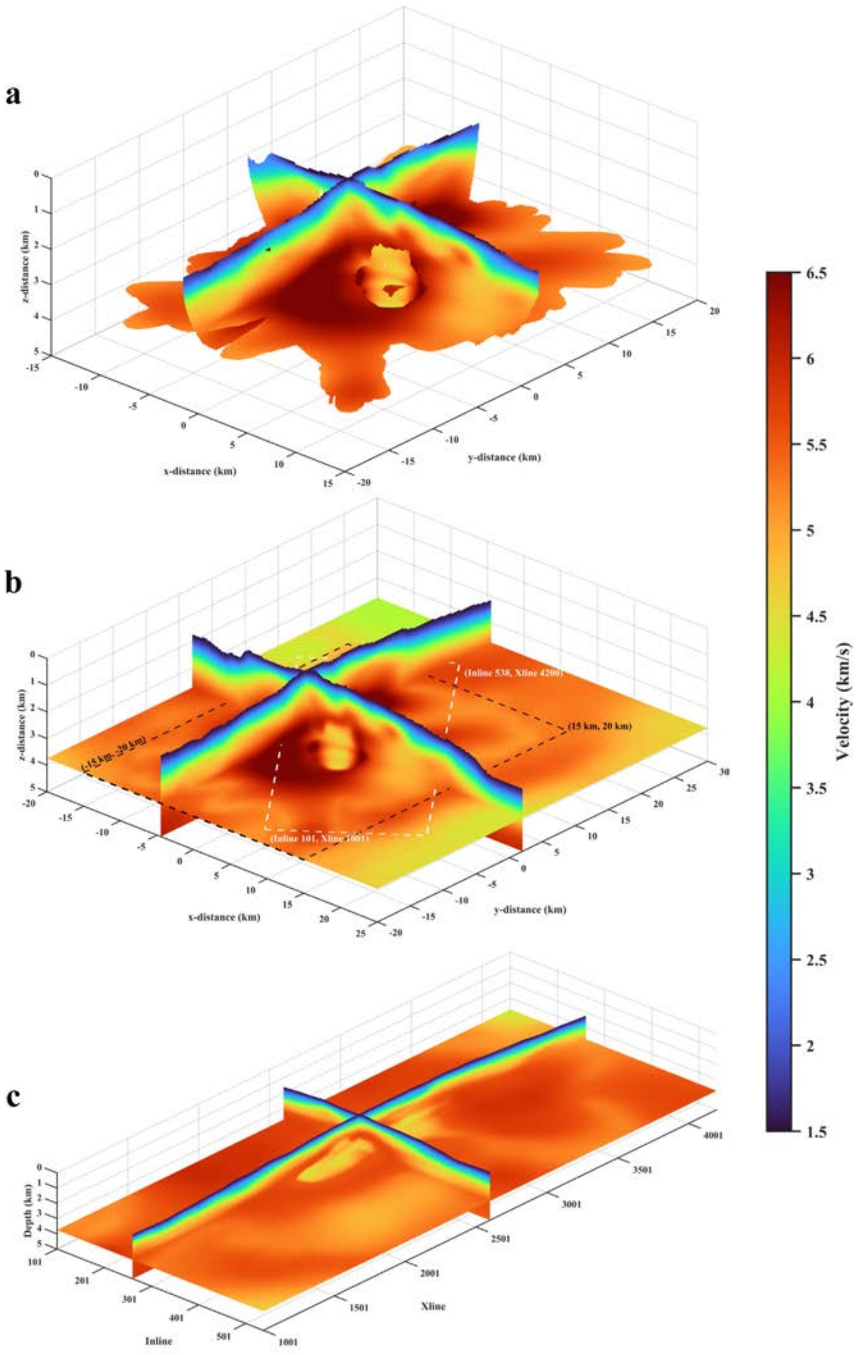

Supplementary Figure 10**. Construction of the initial velocity model. a** The velocity along two crossing vertical profiles and one depth slice at 3.75 km depth from the 3D tomographic velocity model derived by Arnulf et al.[8] **b** Same as **a** after the modification of the tomographic velocity model by gap filling, 3D smoothing, water column velocity adjustment, and out-of-bounds velocity assignment. The black box outlines the original extent of the tomographic model, while the white box marks the survey area of this study. Two velocity models are centered at 45.91792°N and 129.99305°W, and rotated 12.9° to the north. **c** The velocity model along two crossing profiles (Inline 261 and Xline 2600) and depth slice at 3.75 km from the final 3D velocity model extracted and resampled within the study area. The numbers along the horizontal axes are Inline and Xline numbers used throughout this study.

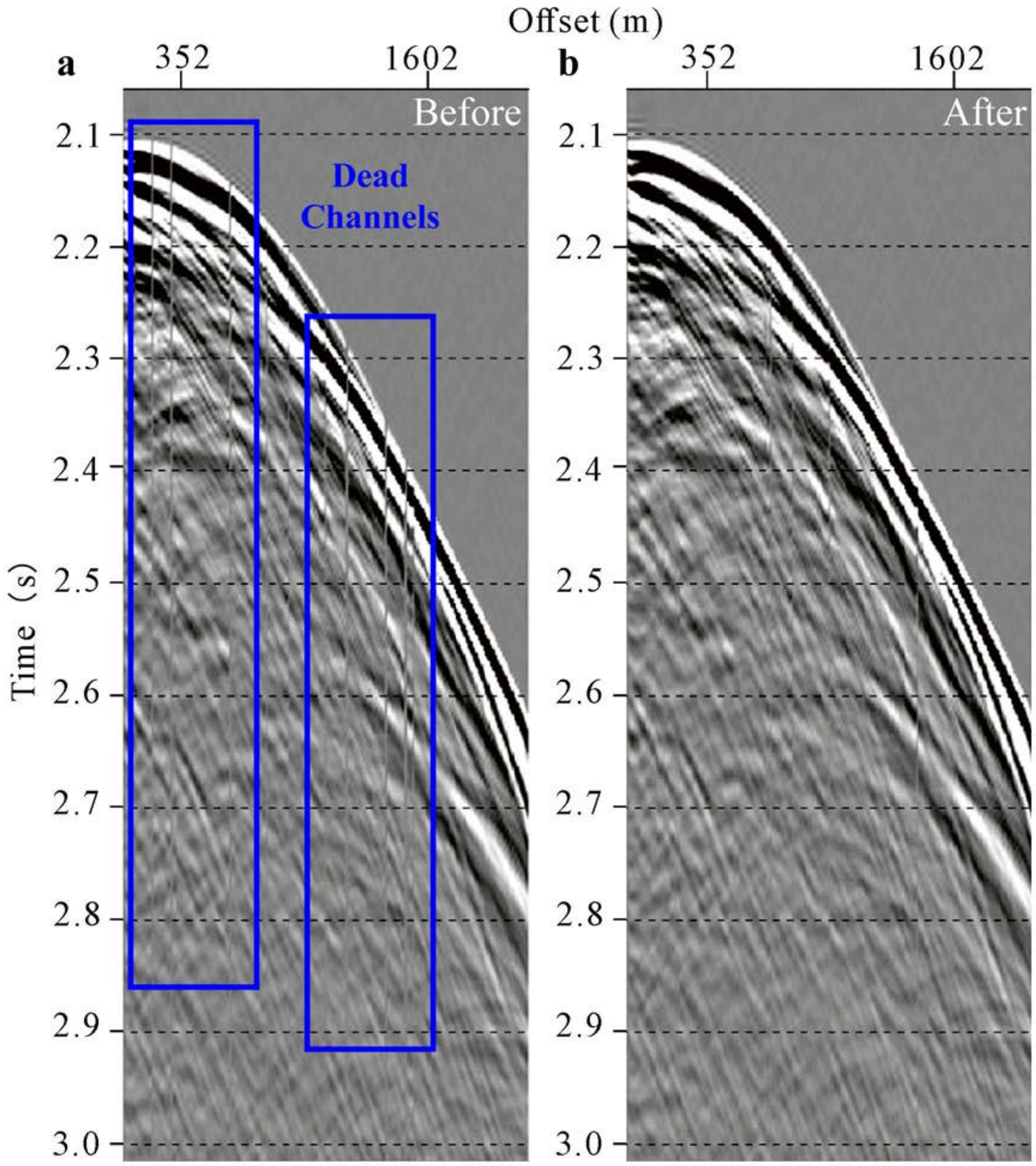

Supplementary Figure 11. **Interpolation. a** Shot gather before and **b** after data interpolation (See Methods). The blue boxes emphasize the presence of dead channels with zero signals.

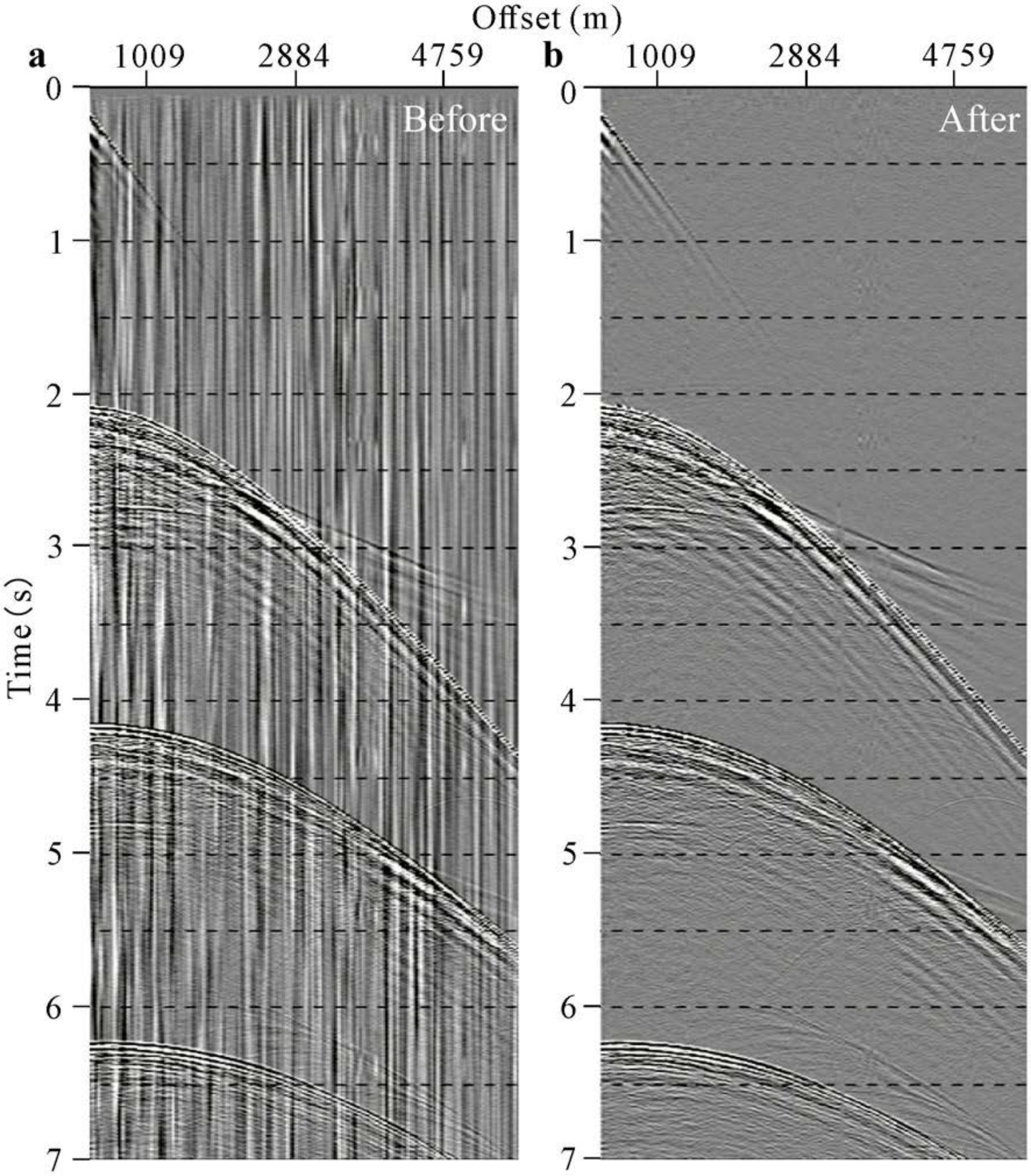

Supplementary Figure 12. **Swell noise attenuation. a** Shot gather before and **b** after swell noise attenuation using the expectation-maximisation algorithm[9].

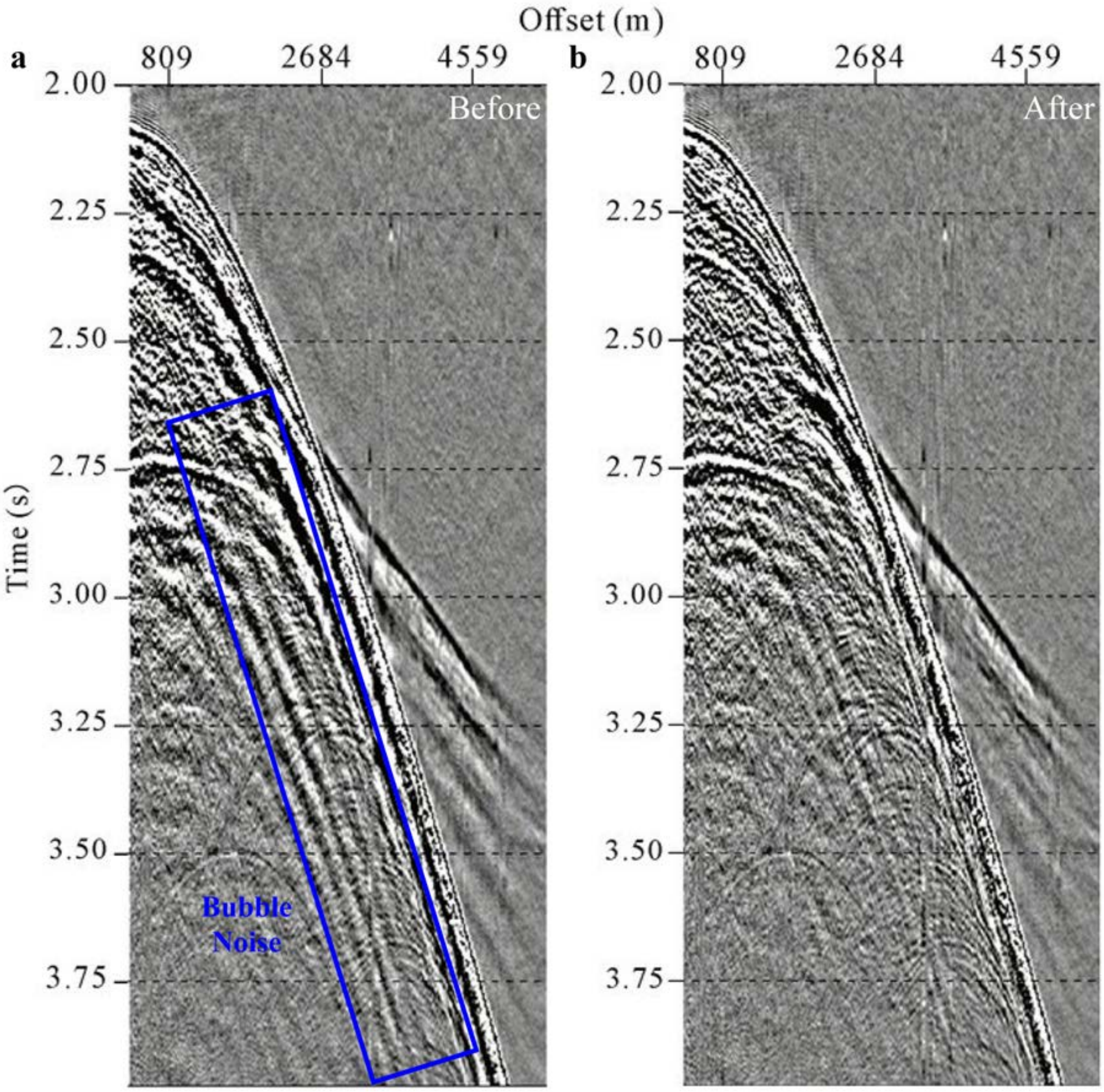

Supplementary Figure 13. **Deconvolution. a** Shot gather before and **b** after deconvolution. The blue box emphasizes the bubble noise in **a** before the deconvolution. After deconvolution, periodic signals due to bubble pulse have been removed.

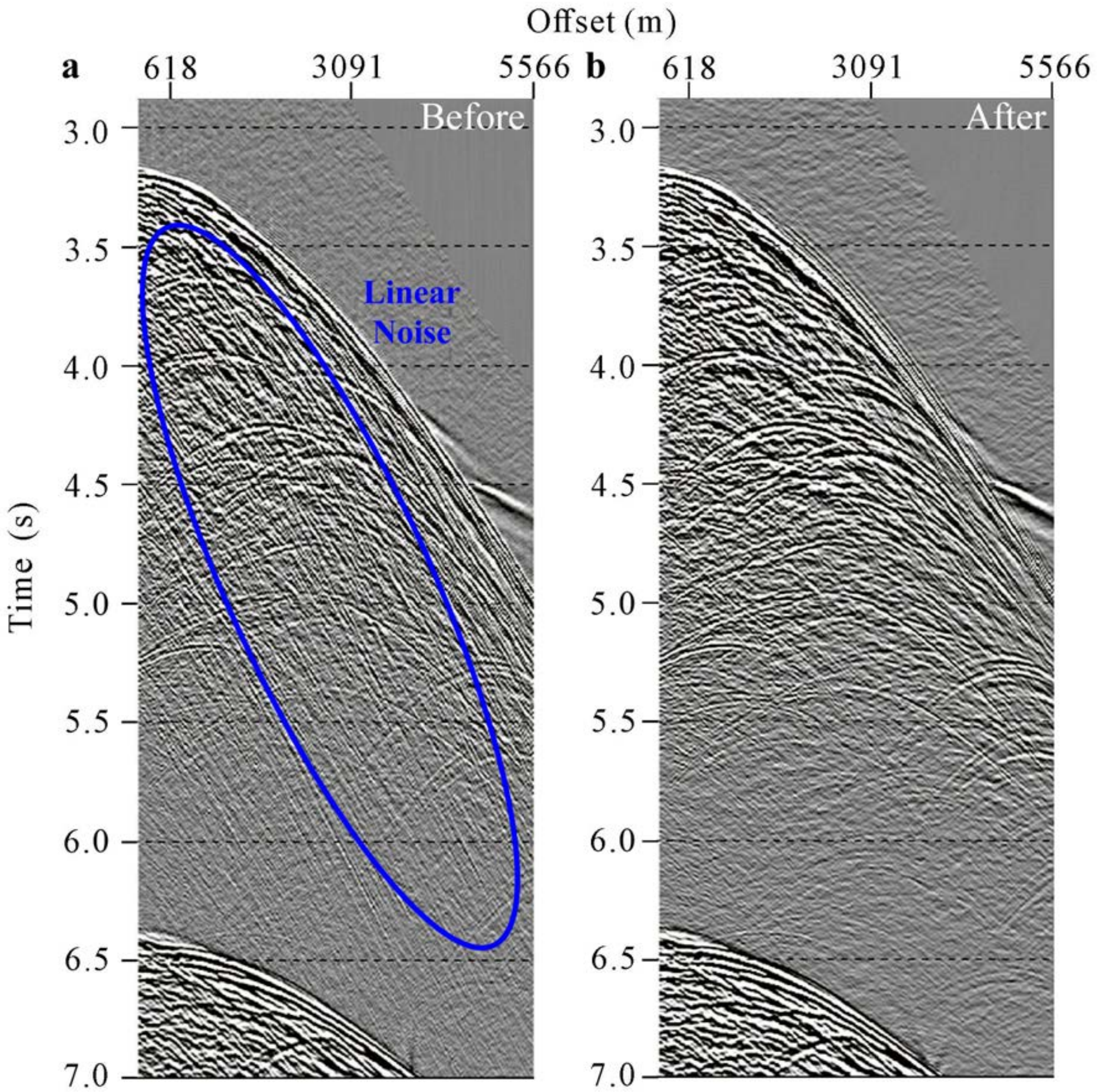

Supplementary Figure 14. **Linear dipping noise removal. a** Shot gather before and **b** after linear dipping noise removal. The blue ellipse emphasizes the linear dipping noise in **a**, which have been removed after the dip filter (see Methods).

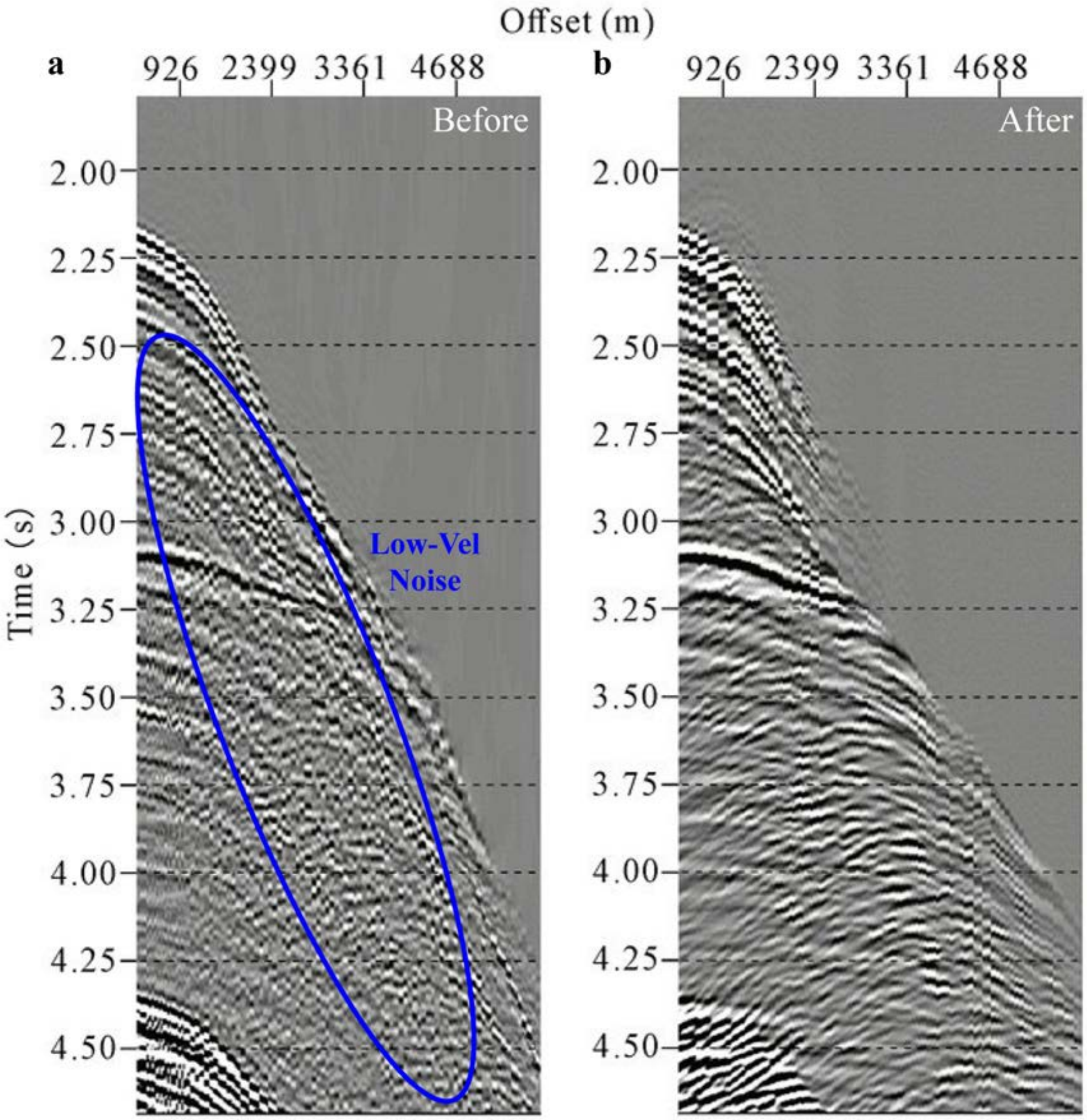

Supplementary Figure 15. **Low velocity energy suppression.** **a** Common-midpoint (CMP) gather before and **b** after suppression of energy travelling with low-velocities (<1.8 km/s). The blue ellipse emphasizes the low velocity noise.

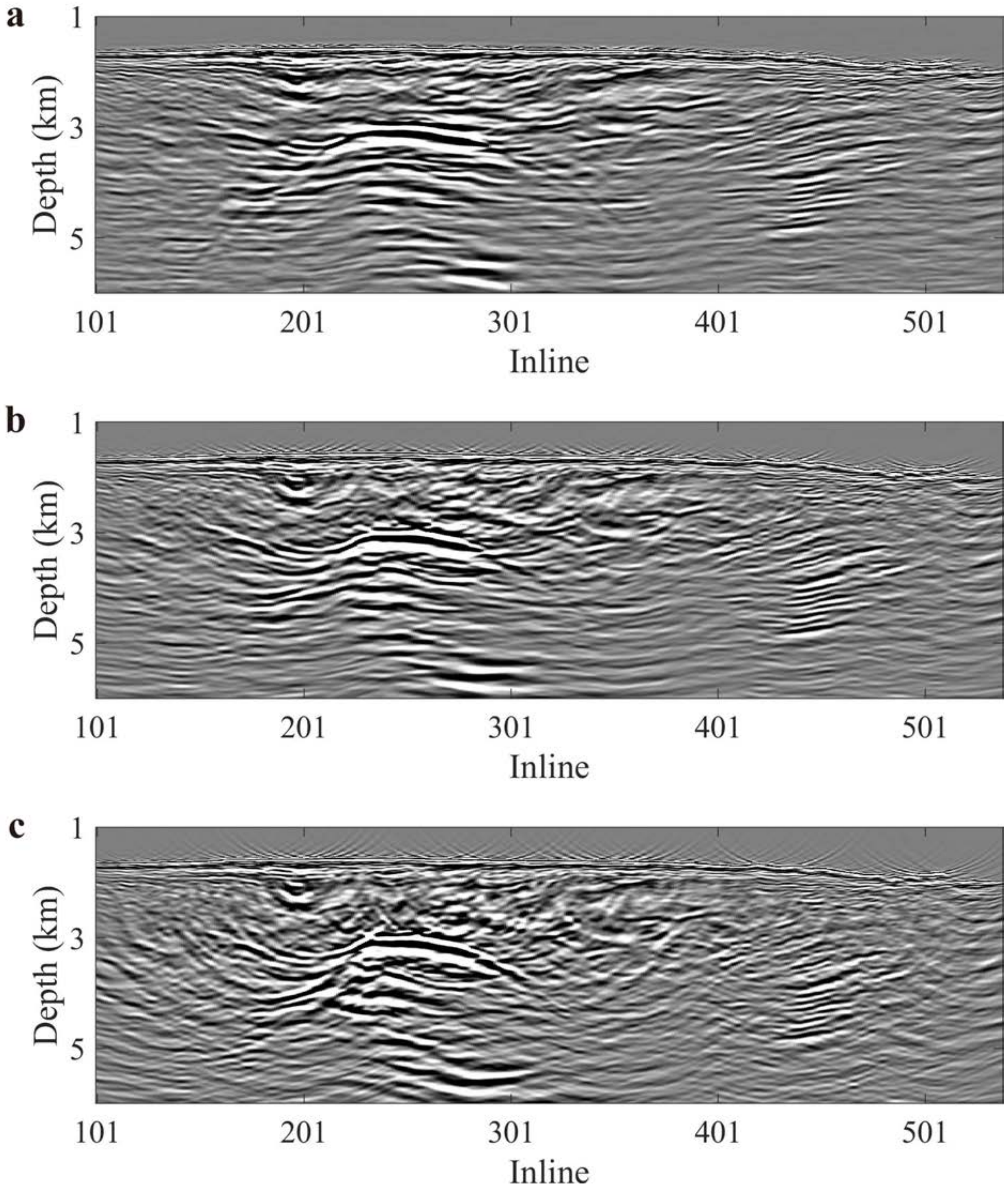

Supplementary Figure 16. **The effect of aperture on 3D prestack depth migration (PSDM).** A comparison of seismic images using different migration apertures in the Xline direction along Xline 2424. **a** Image using 1 km aperture, **b** image using 2 km aperture, **c** image using 4 km aperture. The 4-km aperture PSDM produces sharpest image of the magma domain and its dipping flanks but produces migration artefacts (smiles) reducing clarity of the lava flow layering in the shallow part. The 1-km aperture PSDM produces clear image of the upper crustal layering but produces migration artifacts (smearing).

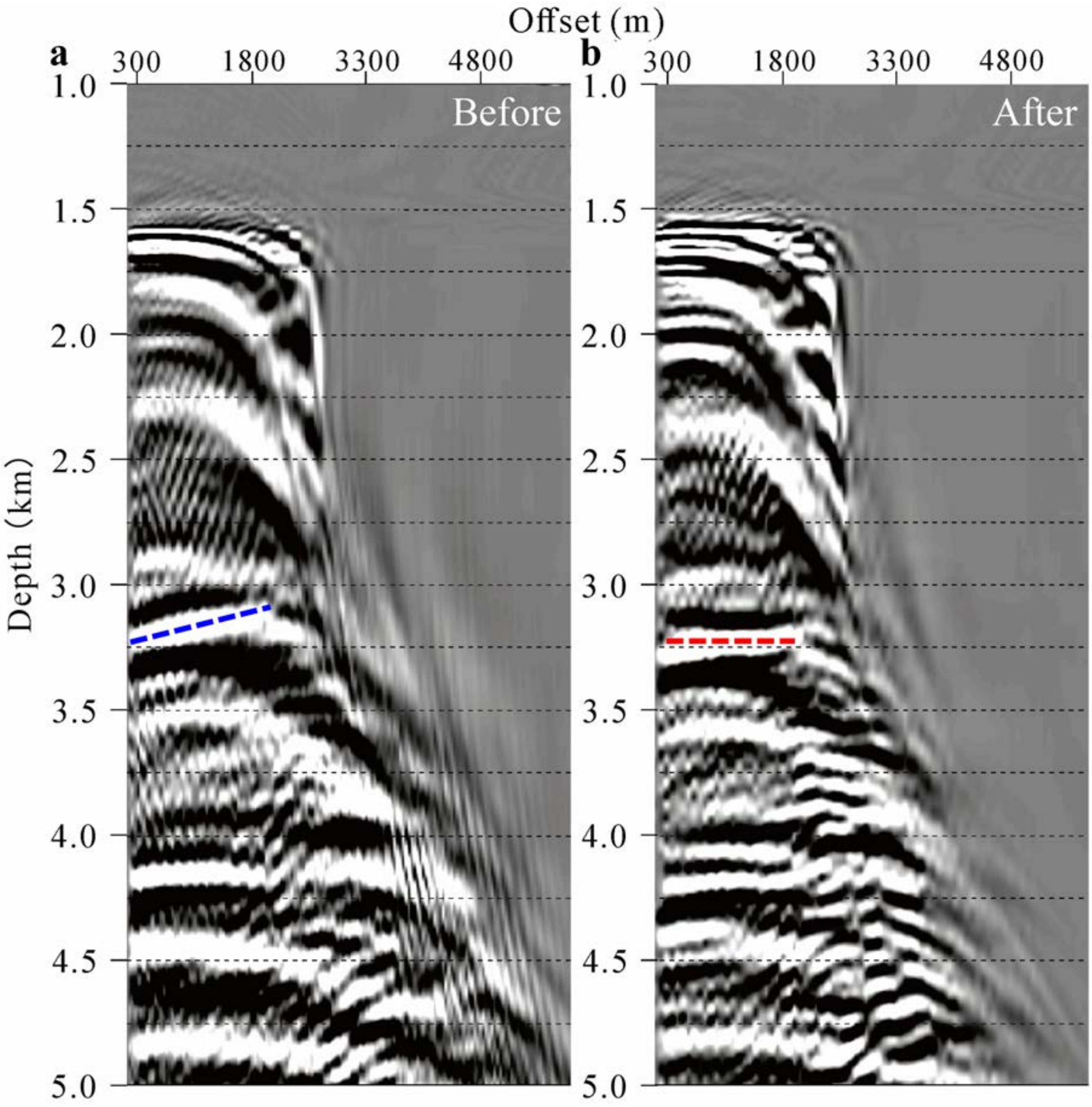

Supplementary Figure 17. **Residual Moveout (RMO) Correction. a** Common imaging gather (CIG) before and **b** after the RMO correction. The blue and red dotted lines indicate the slopes of the melt sill reflection event before and after the correction. The horizontal event in **b** (red) indicates the migration velocity is accurate enough to flatten the CIGs.

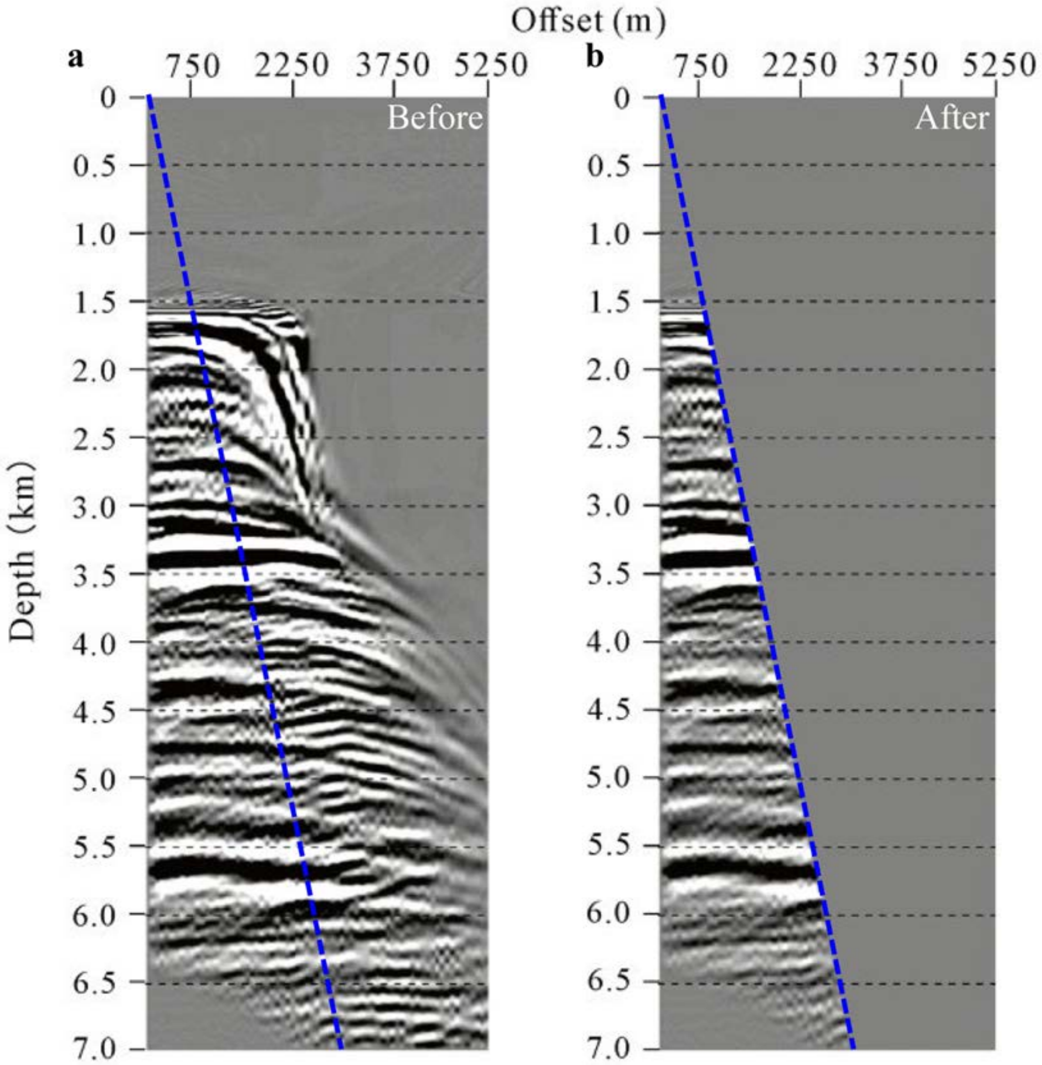

Supplementary Figure 18**. Common imaging gather (CIG) muting. a** Common imaging gather (CIG) before and **b** after mute. The blue dotted lines indicate the boundary of the mute function. Only data left of the blue line are used for producing images shown in this study.

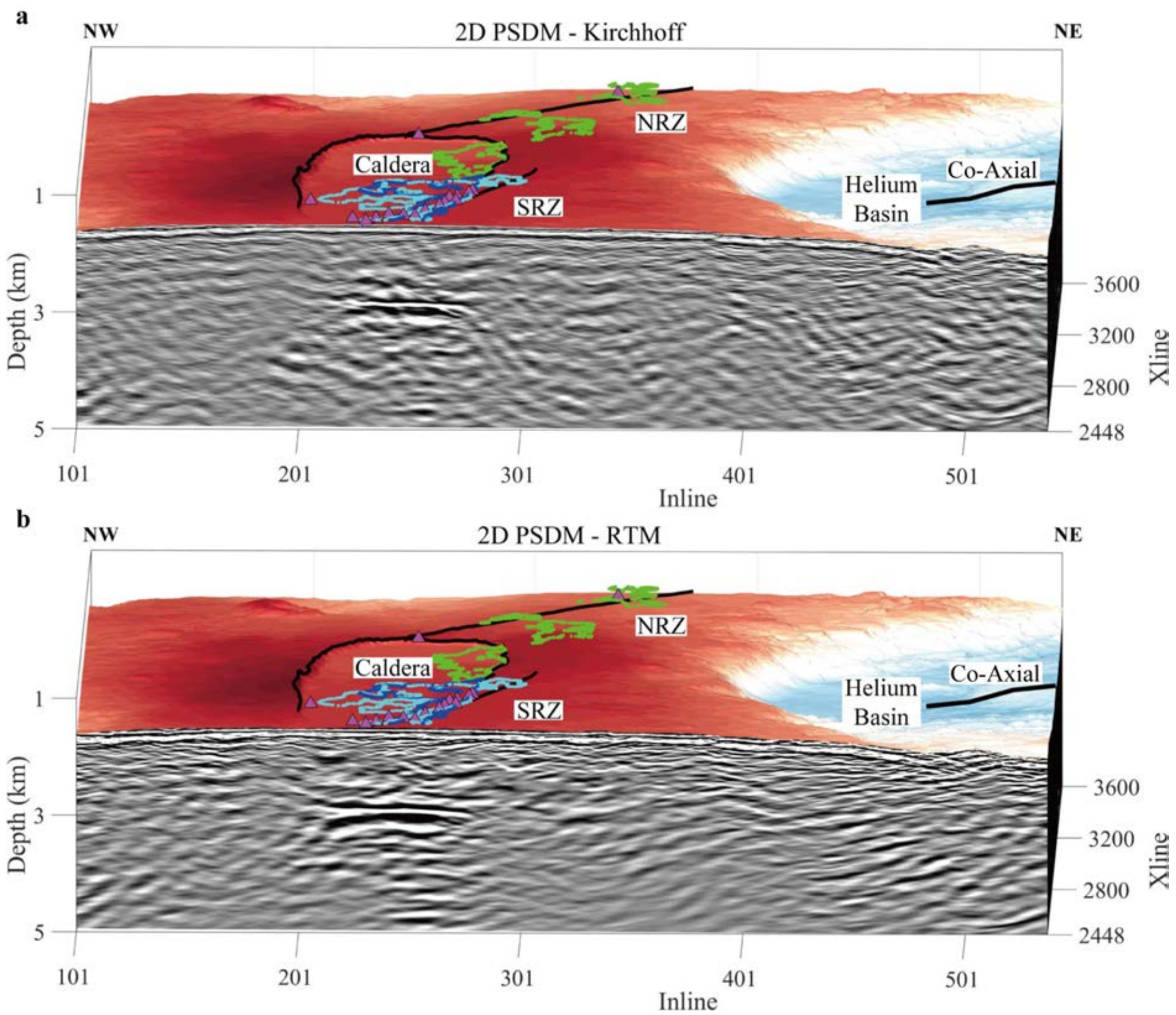

Supplementary Figure 19. **Kirchhoff versus reverse time migration (RTM). a** 2D Pre-stack Kirchhoff migration and **b** 2D reverse time migration (RTM) of 2D profile 2003, which is coincident with Xline 2448.

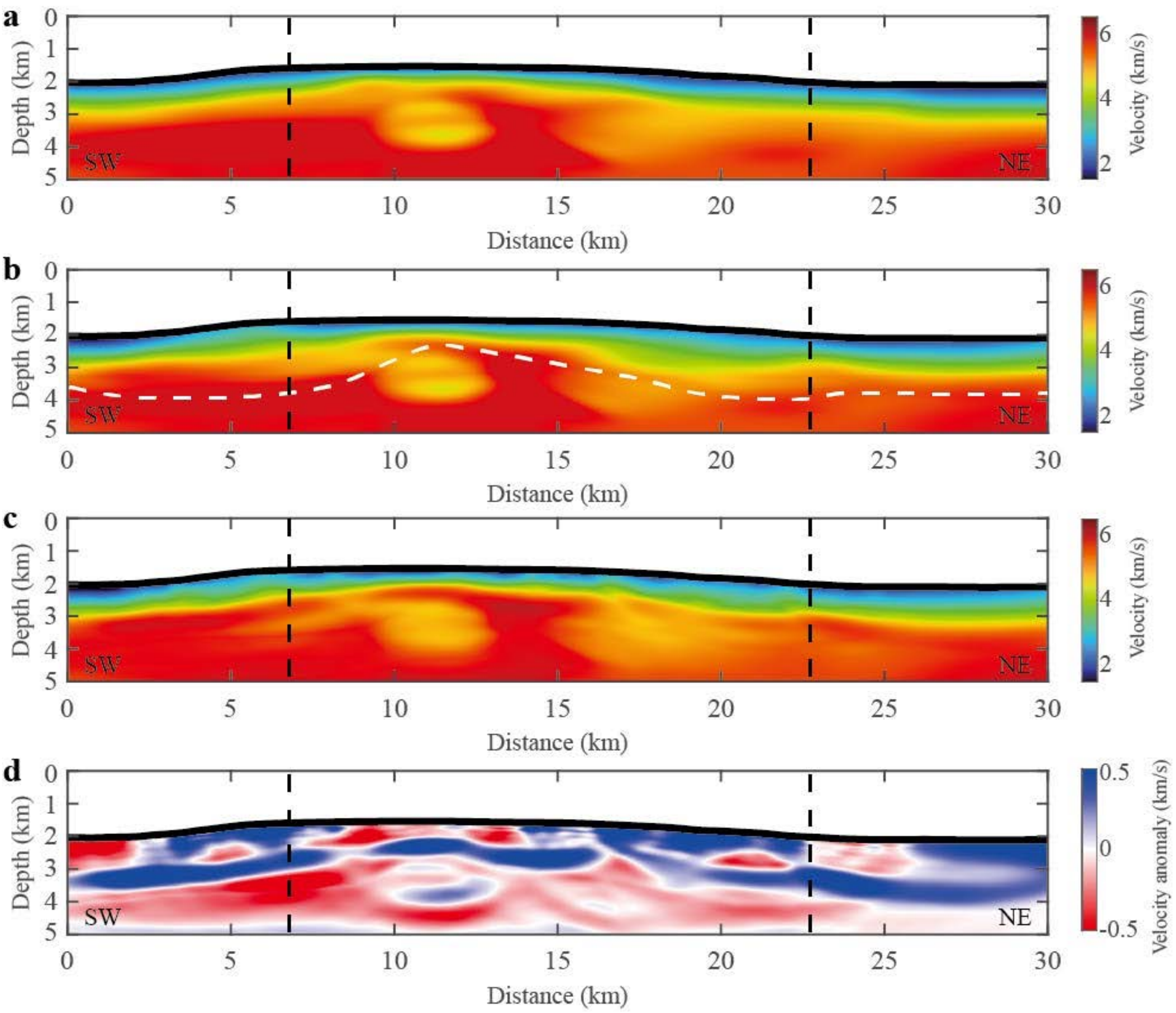

Supplementary Figure 20. **Full waveform inversion (FWI) and velocity models. a** The initial velocity model for tomographic inversion along 2D profile 2003, which also corresponds to Xline 2448. **b** The inverted model produced by travel time tomography; the white dash line indicates the maximum depth of ray coverage. **c** The inverted model produced by FWI using the velocity in **b** as the starting model. **d** Velocity updates to the initial model after tomography and FWI. The black dashed lines indicate the distance range in which the 1D velocity extracted and shown Supplementary Figure 8.

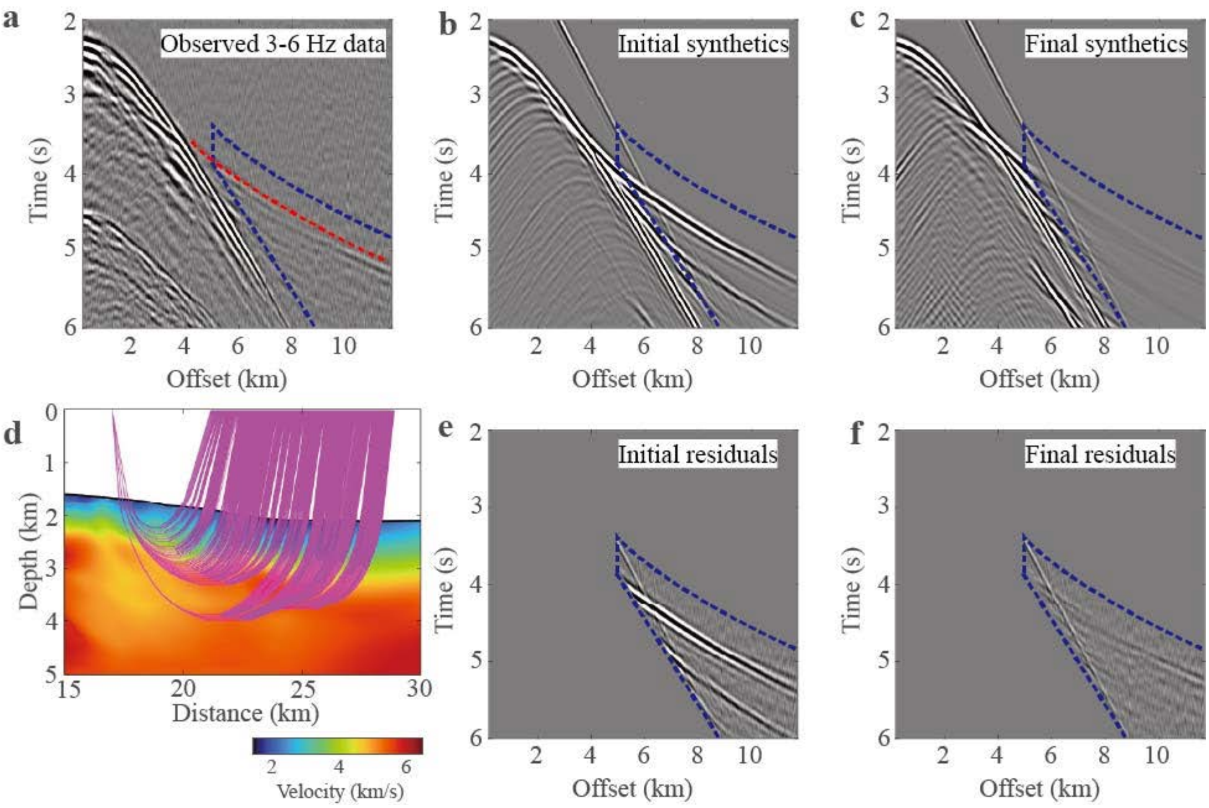

Supplementary Figure 21**. An Example of 2D seismic reflection data waveforms and residuals. a** Shot gather No. 1229 at 17 km distance along the 2D profile 2003. The red dashed line indicates the travel time picked from the wide-angle first arrivals for travel time tomography. The blue dashed lines mark the data window used for full waveform inversion (FWI). **b** Synthetic data computed from the tomographic velocity model, which was the starting model for the FWI. **c** Synthetic data computed from the final model obtained using FWI. **d** The location of the shot gather and corresponding rays in the final velocity model. **e** Data residual between initial synthetic data and observed data. **f** Data residual between final synthetic data computed after FWI and observed data.

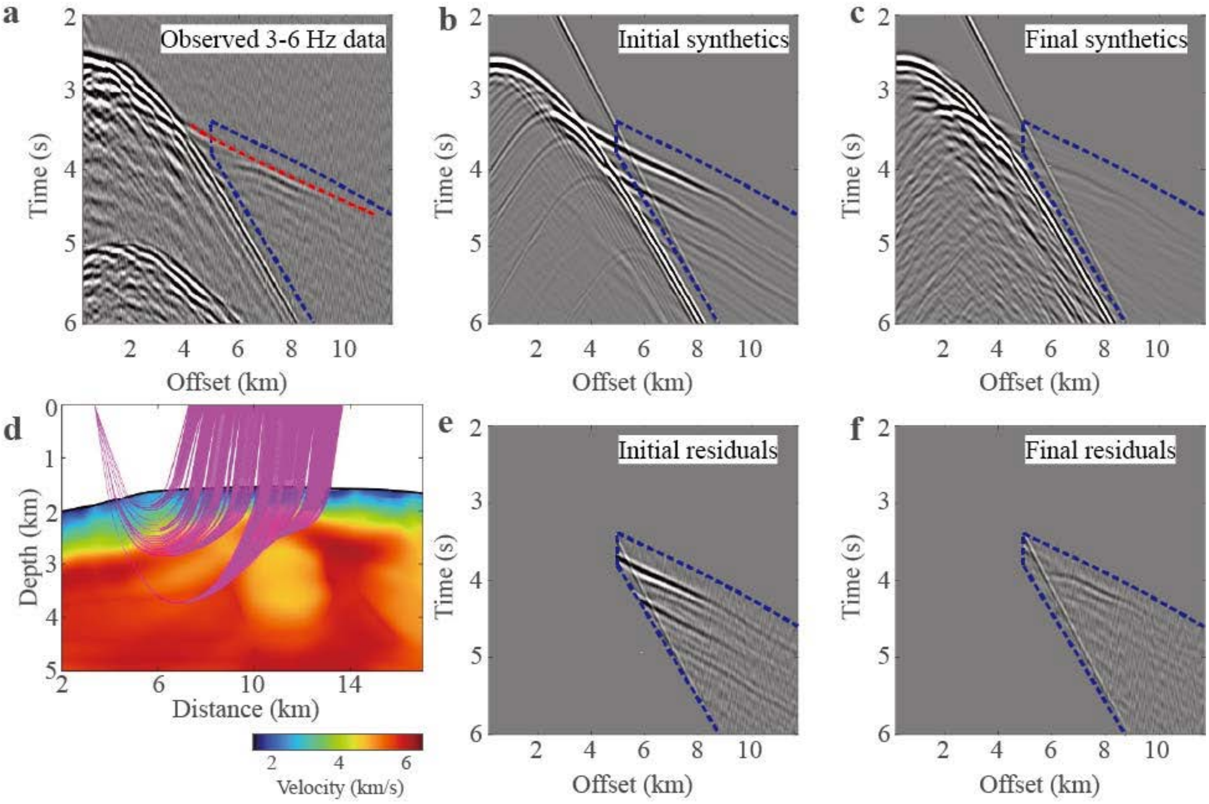

Supplementary Figure 22**. An Example of 2D seismic reflection data waveforms and residuals. a** Shot gather No. 1593 at 3 km distance along the 2D profile 2003. The red dashed line indicates the travel time picked from the wide-angle first arrivals for travel time tomography. The blue dashed lines mark the data window used for full waveform inversion (FWI). **b** Synthetic data computed from the tomographic velocity model, which was the starting model for the FWI. **c** Synthetic data computed from the final model obtained using FWI. **d** The location of the shot gather and corresponding rays in the final velocity model. **e** Data residual between initial synthetic data and observed data. **f** Data residual between final synthetic data computed after FWI and observed data.

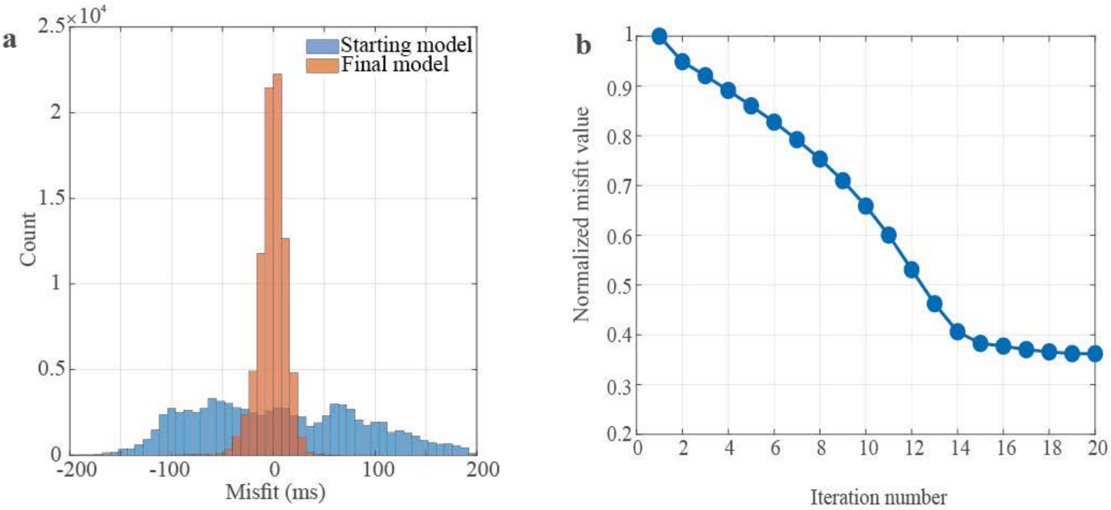

Supplementary Figure 23. **Misfit variation during velocity inversions. a** The histogram of picked travel time difference between the initial and final velocity models for the tomography. **b** The normalised misfit values at each iteration of FWI.

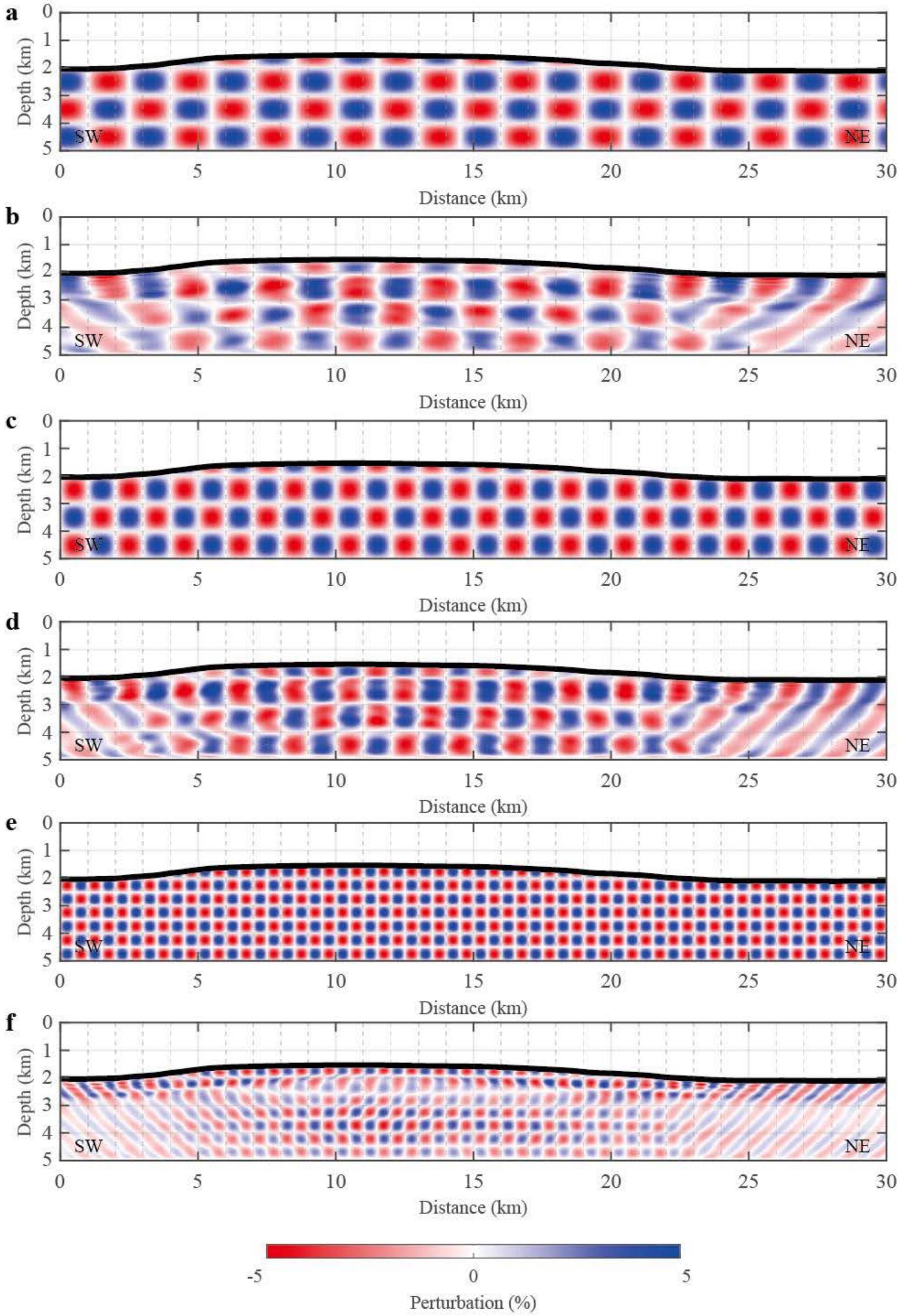

Supplementary Figure 24. **Checkerboard test for full waveform inversion (FWI).** Checkerboards of sizes **a** 1.5 km x 1 km, **c** 1 km x 1 km, and **e** 0.5 km x 0.5 km with a

perturbation of ±5% in the smoothed version FWI velocity model and the corresponding recovered velocity models **b, d,** and **f** after FWI, respectively. The perturbation is of sinusoidal in nature.

Reference

1. Baker, E. T. *et al.* Posteruption Enhancement of Hydrothermal Activity: A 33-Year, Multieruption Time Series at Axial Seamount (Juan de Fuca Ridge). *Geochem. Geophys. Geosystems* **20**, 814–828 (2019).
2. Butterfield, D. A., Massoth, G. J., McDuff, R. E., Lupton, J. E. & Lilley, M. D. Geochemistry of hydrothermal fluids from Axial Seamount hydrothermal emissions study vent field, Juan de Fuca Ridge: Subseafloor boiling and subsequent fluid-rock interaction. *J. Geophys. Res. Solid Earth* **95**, 12895–12921 (1990).
3. Caress, D. *et al.* Interpreted outlines (version 1) as ASCII points of the 2011 lava flows and eruptive fissures at Axial Seamount, Juan de Fuca Ridge (investigator David Caress). Interdisciplinary Earth Data Alliance (IEDA) https://doi.org/10.1594/IEDA/323602 (2018).
4. Chadwick, W. *et al.* Interpreted outlines as ASCII points of the 1998 lava flows and eruptive fissures at Axial Seamount, Juan de Fuca Ridge (investigator William Chadwick). Interdisciplinary Earth Data Alliance (IEDA) https://doi.org/10.1594/IEDA/323601 (2018).
5. Clague, D. *et al.* Interpreted outlines (version 2) as shapefiles of the 2015 lava flows and eruptive fissures at Axial Seamount, Juan de Fuca Ridge (investigator David Clague). Interdisciplinary Earth Data Alliance (IEDA) https://doi.org/10.1594/IEDA/324417 (2018).
6. Kent, G. M. *et al.* Melt focusing along lithosphere–asthenosphere boundary below Axial volcano. *Nature* **641**, 380–387 (2025).
7. Taylor, M. A. J. & Singh, S. C. Composition and microstructure of magma bodies from effective medium theory. *Geophys. J. Int.* **149**, 15–21 (2002).
8. Arnulf, A. F., Harding, A. J., Kent, G. M. & Wilcock, W. S. D. Structure, Seismicity, and Accretionary Processes at the Hot Spot-Influenced Axial Seamount on the Juan de Fuca Ridge. *J. Geophys. Res. Solid Earth* **123**, 4618–4646 (2018).
9. Bekara, M. & Van Der Baan, M. High-amplitude noise detection by the expectation-maximization algorithm with application to swell-noise attenuation. *Geophysics* **75**, V39–V49 (2010).
