# Supplementary material for: Oceanic upper crustal accretion by melt sill and lava flow interaction at Axial volcano
Source: Nat Commun. 2026 Mar 5;17:3512. doi: 10.1038/s41467-026-70033-x (PMC13084056; doi:10.1038/s41467-026-70033-x)
Supplement: Supplementary file 2 — Description of Additional Supplementary File [file 41467_2026_70033_MOESM2_ESM.pdf]

Description of Additional Supplementary Files

Supplementary Movie 1:

Inline profiles from 101 to 538. 3D movie showing images of Inlines starting from Inline 101 (the first image) at southwest of the 3D box (Fig. 2) at an increment of 37.5 m. The caldera boundary and rift zones are marked with black lines. Locations of hydrothermal fields are shown as small, magenta-filled triangles[1,2]. Outlines of the 1998 (blue), 2011 (cyan) and 2015 (green) eruption lava flows are overlain on the bathymetric map[5]. The movie shows melt sills, lava flow layers and magma domain.

Supplementary Movie 2:

Xline profiles from 1001 to 4200. 3D movie showing images of Xlines starting from Inline 1001 (the first image) at southwest of the 3D box (Fig. 2) at an increment of 12.5 m. The caldera boundary and rift zones are marked with black lines. Locations of hydrothermal fields are shown as small, magenta-filled triangles[1,2]. Outlines of the 1998 (blue), 2011 (cyan) and 2015 (green) eruption lava flows are overlain on the bathymetric map[3-5]. The movie shows melt sills, lava flow layers and magma domain.

Supplementary Movie 3:

Three-dimensional rotating Figure 3 with lava flow layer base surface. A movie showing rotating (360º) Figure 3 (Supplementary Figure 2) with the 3D surface highlighting base of the lava flow layer with red (shallow) and blue deep. NRZ: North rift zone. SRZ: South rift zone. The caldera boundary and rift zones are marked with black lines. Locations of hydrothermal fields are shown as small, magenta-filled triangles[1,2]. Outlines of the 1998 (blue), 2011 (cyan) and 2015 (green) eruption lava flows are overlain on the bathymetric map[3-5]. The movie highlights lava flow layering and their relationship with melt sills. Colour surface represents one of the prominent dipping flow layers mapped in the 3D data volume, from shallow (red) to deep (blue).

Supplementary Movie 4:

Three-dimensional rotating Figure 4 with lava flow layer base surface. A movie showing rotating (360º) Figure 4 (Supplementary Figure 4) with the 3D surface highlighting base of the lava flow layer with red (shallow) and blue (deep). NRZ: North rift zone. SRZ: South rift zone. The caldera boundary and rift zones are marked with black lines. Locations of hydrothermal fields are shown as small, magenta-filled triangles[1,2]. Outlines of the 1998 (blue), 2011 (cyan) and 2015 (green) eruption lava flows are overlain on the bathymetric map[3-5]. The movie highlights 3D nature of lava flow layering and their relationship with melt sills. The blank (white) area represents boundary of

caldera at depth. Colour surface represents one of the prominent dipping flow layers mapped in the 3D data volume, from shallow (red) to deep (blue).

Supplementary Movie 5:

Three-dimensional rotating Figure 7 with lava flow layer base surface. A movie showing rotating (360º) Figure 7 (Supplementary Figure 6) with the 3D surface highlighting base of the lava flow layer with red (shallow) and blue (deep). NRZ: North rift zone. SRZ: South rift zone. The caldera boundary and rift zones are marked with black lines. Locations of hydrothermal fields are shown as small, magenta-filled triangles[1,2]. Outlines of the 1998 (blue), 2011 (cyan) and 2015 (green) eruption lava flows are overlain on the bathymetric map[3-5]. The movie highlights 3D nature of lava flow layering and their relationship with melt sills. The blank (white) area represents boundary of caldera at depth. Colour surface represents one of the prominent dipping flow layers mapped in the 3D data volume, from shallow (red) to deep (blue).
